# Integrating Key Nursing Measures into a Comprehensive Healthcare Performance Management System: A Tuscan Experience

**DOI:** 10.3390/ijerph19031373

**Published:** 2022-01-26

**Authors:** Chiara Barchielli, Anne Marie Rafferty, Milena Vainieri

**Affiliations:** 1 Management and Health Laboratory, Institute of Management, Sant’Anna School of Advanced Studies, 56127 Pisa, Italy; milena.vainieri@santannapisa.it; 2 Midwifery & Palliative Care, Florence Nightingale Faculty of Nursing, King’s College London, London SE1 8WA, UK; anne_marie.rafferty@kcl.ac.uk

**Keywords:** nursing, quality, safety, evaluation, integration, focus group, consensus process

## Abstract

This paper addresses the evaluation of nursing quality and safety beyond nursing tasks in specific healthcare settings and sets it in a context that conveys the sense of complexity and multifaceted nature of the contribution that nursing makes to the whole system. The paper describes research conducted in Tuscany during 2019 involving regional managers and heads of nursing departments. This research has led to the development of an integrated evaluation framework through focus groups and consensus process with the latter, which includes Performance Organizational climate data, Patient-Reported Experience Measures (PREMs), and Patient-Reported Outcome Measures (PROMs). This integrated framework aims at both making sense of extant measures as key performance indicators shared among different professionals while recognizing the important role of nursing care by adding specific measures and can be seen as a tool that boosts the sense of “teamness” in healthcare.

## 1. Introduction

Since 2000, New Public Management reforms have ushered in a variety of changes and innovations into different parts of the public sector [1,2] including healthcare, propelling the development of multidimensional performance management systems [3]. Given the complexity of healthcare, performance evaluation tools have been designed to measure and monitor different dimensions, such as (i) health outcomes, (ii) health service access, (iii) efficiency, and (iv) service quality and appropriateness [4,5]. Motivations for measurement and assessment in the public sector are several and extensively reported in the literature. For instance, Behn [6] proposed eight reasons for measuring performance: evaluating, controlling, budgeting, motivating, promoting, celebrating, learning, and improving. When measures are used to evaluate, they become elements that help managers and policymakers to make decisions. Indeed, performance evaluation estimates “the quality of health services with the ultimate goal of improving health outcomes” [7]. This is particularly relevant when the performance evaluation system is based on solid measures from administrative and survey data [8] that can help select strategies to continuously improve care and accountability towards citizens [9,10]. Although many scholars uphold the importance of designing and implementing a performance evaluation system at different governance levels [8,9,10,11], there are several factors to be considered (different organizational models, financing mechanisms, governance and resources in service provision [11], stakeholders ‘perspectives, uncertainty, and organizational fragmentation [12]), all of which make performance evaluation in the healthcare field particularly complex. To cope with this complexity, performance evaluation systems in healthcare are characterized by multiple dimensions; quality, efficacy, and responsiveness appear to be the most frequently used dimensions to evaluate performance both at the organizational level [13,14,15] and healthcare pathway, although there are very few examples of visual representation of evaluation system along the entire patient pathway [16].

Within the general field of performance evaluation in healthcare, there is a considerable amount of literature on performance evaluation in nursing. Nursing Sensitive Indicators (NSIs) are the measures used to assess the nursing quality and safety outcomes. In 1996, the American Nurse Association identified NSIs as “those indicators that capture care or its outcomes most affected by nursing care” [17]. The National Quality Forum (2014) stated that NSIs are “a nursing-sensitive performance measure of process and quality and safety outcome and structural proxies for these process and outcomes (e.g., skill mix and nurse staffing hours), that are affected, provided, and/or influenced by nursing personnel, but for which nursing is not exclusively responsible” [18]. Moreover, according to Krau [19], these indicators reflect practice models through which nursing care is organized and provided. It is suggested that NSIs could be used for continuous benchmarking to improve the healthcare system [20]. The development of NSIs allows nurses to manage and control nursing activity and processes. In addition, they enable decisions to be taken with autonomy and appropriateness [21].

Traditionally, NSIs are grouped according to the Donabedian’s classification: “structure, process and outcome framework” [22], but there is also debate over these as Heslop and colleagues [19] argue that NSIs mostly deal with structural attributes related to health services (e.g., hours of nursing care per patient per day and nursing staffing) and with outcomes related to patient care (e.g., the prevalence of pressure ulcers, falls and falls with injury, nosocomial selective infection and patient/family satisfaction with nursing care) that is using NSIs with a solid referral to a nursing conceptual framework. In our study, we underline that NSIs focus on quality as well (safety, clinical management, use of health care, and functional status), satisfaction (perception), and setting (related to health organization) indicators. However, Needleman et al. [23] highlight the importance of the dynamism involved in measurement, suggesting four dimensions: (i) Nursing Practice Environment (NPE), (ii) nurses’ education level and skills, (iii) hospital structure/setting, and (iv) nurse organization (e.g., assistance models) [23]. Within Needleman et al.’s model, outcome measures align with essential organizational factors and human resource management. Significantly, the approaches referred to above are predominantly North American contexts in which nursing has an established and influential role, whereas, in Italy, the potency of nurse leadership is still evolving [24]. A clear example of the different roles played by nursing in Italy is the ratio of physicians and nurses per 1000 population. As the OECD Report 2020 details [25], while Italy is below the nurse-to-population OECD’s average ratio (5.7 on an average value of 8.2), it is above the physician-to-population OECD’s average ratio (4 on an average value of 3.4). The OECD points to the impact the dearth of nurses has on reducing system resilience: nursing expertise should play a more prominent role in clinical and policy decision making. In general, the views of the experts should be taken into account, as for example, the ones from the EXPH—expert panel on effective ways of investing in health [26]—that encourages the introduction of greater flexibility in the system, as the system will rely on nurses more and more, advising to consider the task-shifting as a possible solution to meet people’s health needs. Moreover, as the pandemic has made clear, the need to invest in primary and community care and nursing is central for the health of communities themselves [27,28]. In 2019, the Italian Ministry of Health stated that [29] Italy must invest in the “Family and Community Nurse” care delivery model to be closer to the population through a professional who should not only be a provider of isolated treatments but a general reference figure for the health, prevention, and managing of families and the communities’ health needs. This position was confirmed and reinforced by laws that were enacted during the pandemic time [30], and it has been supported through the healthcare strategies until financed by the “next-generation UE” funds. In this evolving context, it is more urgent than ever to give nursing’s impact on the health system strength and visibility. Hence, the impetus for integrating nursing in a broader framework on healthcare quality and safety came from the need to make the system aware of the role played by nursing into the whole healthcare performance but also to make nurses aware of the fact that they are an integral part of the system, where their effectiveness and accountability can be enhanced.

**Figure 1:**
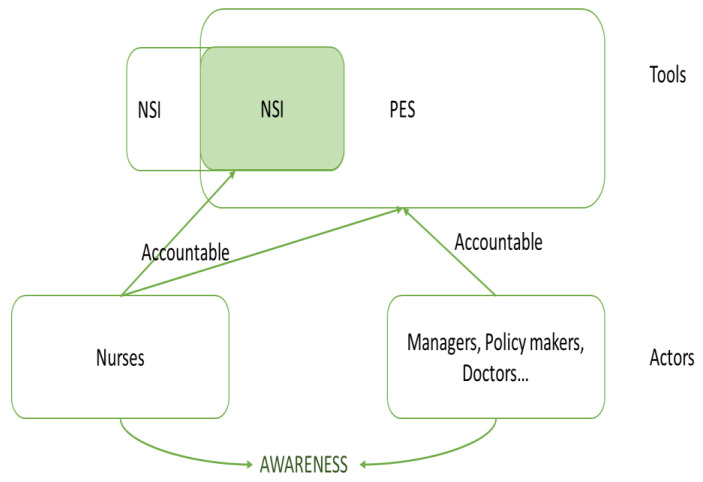
The “awareness virtuous circle”, authors’ conceptualization.

This paper presents a performance model where nursing quality and security (Q&S) indicators are integrated into an existing framework on healthcare system performance evaluation to demonstrate the multifaceted nature of the nursing contribution to the whole healthcare system and to give visibility to nursing management. The Performance Evaluation System (PES) framework was developed in an Italian Regional healthcare system (RHS) in the early 2000s [16,31] and benchmarks all the organizations that take part in it. The integration of nursing indicators is instrumental in two ways: (1) making nurses accountable in the eyes of the system and (2) make the latter aware of how their work can improve Q&S through feedback from the system-heightened awareness as its main engine of improvement, as shown in Figure 1.

The paper describes the constructive research, an approach used to define and solve problems [32] carried out in Tuscany to include NSI into PES and to make sense of the PES for nurses.

## 2. Materials and Methods

Research in health management has the goal of building a theory, a solution, a tool to address a real need and improve an existing system in terms of design and or performance [31,32]. Our study aimed to build a performance evaluation system that would be useful to nurses and capable of integration into an existing system based on routine administrative and survey data. The Tuscan RHS designed and implemented a multidimensional PES in 2014 [31]. To date, PES contains over 700 indicators [1,33] and has spread to other Italian Regions and their RHSs. Through a benchmarking process, it shed evidence on crucial dimensions like population’s health status, health assessment, ability of the healthcare organizations to follow set regional health strategies, evaluation of the experience of users and employees, and operational efficiency [16]. Nursing was not involved in its original design as a stand-alone profession, and our proposal of an adapted PES version that includes nursing performance measures has a dual intent: (a) make nurses aware of their impact on the system, empowering them to seek improvement and being accountable at a system level; (b) make the system aware of the nursing role in achieving better outcomes.

First, we surveyed the existing literature on nursing quality and safety systems to inform our subsequent focus group with Directors of Nursing Departments in Tuscany that were formally and directly recruited explaining the research relevance of the project. Appendix A reports the information according to the consolidated criteria for reporting qualitative research (COREQ).

Due to the estimated impact of the significance of finding more accurate ways to measure nursing quality and safety measures beyond the boundaries of the profession itself to the advantage of a more systemic estimation, all the identified components of the group accepted to be part of the focus group. It lasted about 3 h with the eight Directors of Nursing Departments—all of them experienced Registered Nurses and with seniority of no less than 30 years in the field of the Tuscan RHS, and it was dual-moderated by two of the authors: a Nurse (CB) and an Associate Professor (MV), both with specific knowledge in the fields of nursing, performance evaluation, and management. It was conducted in a formal RHS location, and the participants were introduced to the open discussion by the presentation of compliance guidelines to it. The used interview guide was developed based on an extending study of the existing literature on nursing quality and safety indicators. The overall recorded process led to the identification of the key measures they use in their organizations and found that all the measures identified were comprised in two systems: CaLNOC (Collaborative Alliance for Nursing Outcomes) and NDNQI^®^ (National Database of Nursing Quality Indicators) as more complete, comprehensive, and useful. A description of these two instruments, as well as the PES, are reported in Appendix B to this paper.

Nursing indicators were identified according to a series of criteria: (i) nurses and other health professionals were actively involved in the selection process and the ongoing item review, (ii) clinical trials had validated each indicator [34,35,36,37], (iii) measures linked to whole system performance measurement at a national level, and (iv) indicators that linked to organizational factors and human resources (e.g., nurse to patient ratio). For an in-depth analysis of these themes, see Appendix B. During the focus group with the Directors of Nursing Departments of the Tuscan RHS, the selected nursing indicators to be included in PES have been grouped into four categories: clinical area, regional strategies, patient voice, and human resource management, which are described below.

The second phase consisted in screening indicators included in the CaLNOC [38] and NDNQI^®^ [33] bundles that were already in the PES.

The final phase was the graphical representation together with the consensus received from the Directors of Nursing Departments and from regional and national experts, such as members of the Tuscany Region and the Ministry of Health.

## 3. Results

The focus group and the consensus process generated 56 nursing indicators to highlight nursing within the RHS evaluation process more broadly. The indicators were grouped into four domains, as in Table 1:

1. Clinical domain: nurses, like other healthcare professionals, contribute to the achievement of regional goals like addressing people’s needs in a safe way, providing qualified and attentive help to the customer/patient, etc. In this area, indicators such as the average stay performance index (as a proxy of hospital efficiency) were selected. The information sources are, again, ministerial and regional data, for example, the information flow on hospital discharge records, outpatient services, supplied drugs, etc.

2. Patients’ voice domain: this dimension includes the indicators that derive from patients’ satisfaction and experience (PREMs and PROMs). These patient perception measures have the goal of shaping the healthcare system in a patient-centered way [39,40] by the continuous analysis of the feedback that comes from the end users of the system itself. Through these tools, it is also possible to estimate specific aspects such as the degree of humanization of care and the perceived level of collaboration between the different professionals.

3. Human resource management: this dimension includes both administrative measures like the absence rate and survey data deriving from the Organizational Climate Survey [41] administered every two years in Tuscany from which the indicators on nurses’ job satisfaction, the willingness to recommend, the intention to leave, etc., are selected.

In addition, the selected indicators relate to different settings, such as organizational (firm), hospital, community, and nursing home.

Table 1 lists the indicators that were selected. 

## 4. Discussion

The PES provides the opportunity to expand understanding and action on identified organizational targets to enhance performance improvement. Introducing nursing measures in a consolidated performance measurement system, which is used by policy makers and managers, is a way to ensure that these measures are available to be used by the whole system and not only by one professional category. Moreover, disclosure of data can have positive effects: the process of public sharing activated reputational mechanisms capable of stimulating Health System improvement [42]. Such improvements could result in increasing managerial accountability [43]. On the other hand, there is possible reputational damage that managers could experience otherwise [44,45,46,47]. It has a high visual impact, is easy to understand [48], and is based on a rigorous methodology of collection, processing, and disseminating, providing robust information to track change and benchmarking over time. The indicators can be used to conduct analyses on several levels: national, regional, hospital-based, department-based, nursing home. The level of detail allows strategic, tactical, operational, nurse-specific analysis to be targeted and actions taken. The added value of this enriched framework also lies in the fact that it is co-created with (i) professionals, focusing on their performance but also considering the organizational climate in which they operate and (ii) taking account of patients, to make their point of view the litmus test of the appropriateness of the care provided. The twofold aim that we want to achieve is to highlight the role of nurses within the health system and make it objective in their eyes, thus increasing their motivation [49]; as pointed out at the beginning of this discussion, the aim is, in fact, to motivate, promote, and improve [6] the healthcare systems is of paramount importance. The PES, not only indicators, cover the activities for which nurses are responsible, but all processes in which they participate, to consider their systemic contribution. It also becomes clear that their contribution is integrated with that of other professions, strengthening the position of the need for multi-professionality in healthcare [50]. For their part, the Directors of Nursing Departments of Tuscany that engaged in the focus group did not oppose resistance in the choice of indicators to be accountable for; this is probably because they expressed the will of the whole professional body to be more visible and recognized ad deeply rooted in the various settings of the healthcare system. If the nurses had chosen to use only and only the NSI from CaLNOC and NDNQI^®^, they would have evidenced the will to be accountable in a separate way from all the other professionals. This inclusive and integrative approach was greatly appreciated by the regional and national levels who feared a breakup.

## 5. Conclusions

The present experience, based on the Italian context, can be an inspiration or an example for countries in which organizational models are still based on the role of the physician, as the Italian model is [51]. The pandemic highlighted the importance of Primary Health Care (PHC) [52,53], an approach that follows a person and his/her needs throughout their lifetime and that puts nurses in a central position [54].

This analysis is Tuscany based and reflects the strategies and the characteristics of a single region. The aim is to replicate it in different Italian regions that use PES as an evaluation tool, eventually reaching the national level. Regarding the diffusion of the tool, consideration must be made. Although the PES has been in place for several years, it never encountered a radical, game-changing event like a pandemic, which distorted traditional programming and control processes. We expect a growing role of nursing for the effect of the necessity of a task shifting [26] and for the indispensable expansion of community cares, and we expect PES to reflect it. The ultimate aim of this tool is to offer safe and quality care to patients, and the measures that it provides are meant to inform the managers on the state of the art of the overall process of care itself. When the “awareness virtuous circle” is triggered, a sense of “teamness” moves along, as all the actors of the system can monitor the performance they all contributed to. Furthermore, nurses, physicians, managers, etc., perceive how strong their interdependence along the entire patient pathway is. Managers in particular can enhance their ability to set the way in multiple settings and immediately monitor the effects of their policies and decisions. The result is that the overall performance is enhanced [55].

Although this investigation has benefited from multiple contributions and points of view at different levels, it still needs further considerations and confrontations with professionals from other backgrounds and places. There are still aspects to be discussed and measures to eventually be included, for example, specific metrics on interprofessional teamwork or on specific areas, such as the mental health setting. The state of advancement of this framework strictly reflects the consensus reached on it, and it is open to future changes. One of the possible future improvements is the application of quantitative methods, even very sophisticated like the vector evaluation genetical algorithm (VEGA) that are used to solve real-world optimization problems [56] in cases in which management has to pursue more than one objective and they need to consider multiple factors.

## Figures and Tables

**Table 1:** The 56 nursing indicators’ proposal.

Indicators for Nursing Management	Level of Governance ^1^	Overlapping International Indicators
**Clinical Evaluation**		
1. Index of average length of stay in the medical setting	O	
2. Index of average length of stay in the surgical setting	O	
3. Index of adherence to the RHS bundle of infection and sepsis	O	
4. Index of adherence to hospital handover best practice	O	
5. Hospitalization rate of heart failure per 100,000 residents (50–74 years)	O	
6. Hospitalization rate for diabetes per 100,000 residents (35–74 years)	O	
7. Major diabetes-related amputation rate per million residents (3-year timespan)	O	
8. Chronic obstructive pulmonary disease (COPD) hospitalization rate per 100,000 residents (50–74 years)	O	
9. Percentage of emergency codes: hospitalized/deceased/transferred patients	H	
10. Percentage of Multi Drug Resistance (MDR) infections during hospitalization	O	NDNQI^®^
11. Not self-sufficient patients with pressure ulcers in nursing home	NH	NDNQI^®^
12. Not self-sufficient patients with pressure ulcers in different settings than the nursing homes	NH	NDNQI^®^
13. Pressure ulcers’ clinical improvement for nursing home residents	NH	
14. Patient’s falls occurred in nursing home setting determining access to the Emergency Department (ED), hospitalization, or death	NH	NDNQI^®^ CaLNOC
15. Patients’ falls in nursing home	NH	NDNQI^®^ CaLNOC
16. Nursing home patients with a filled falls risk assessment form	NH	
17. Existence of falls prevention plan in nursing homes	NH	
18. Patients at risk of falls, fallen during the last year in the nursing home setting	NH	
19. Physically restrained, not self-sufficient patients in nursing home settings	NH	
20. Physically restrained (different from movable bed rails), not self-sufficient patients in nursing home setting	NH	
**Patient Experience**		
21. Clarity of information	H	
22. Humanization of care	H	
23. Teamwork	H	
24. Clarity of information at discharge	H	
25. Overall experience assessment	H	
**Employee Voice**		
26. Percentage of responders: organizational climate survey	O	
27. Percentage of absence from work	O	
28. Management evaluation	H	
29. Communication evaluation	H	
30. Training evaluation	H	
31. Intention to leave	O	
32. Patient–nurse relationship	H	
33. Territorial opioids consumption	O	
**Regional Strategies**		
34. Standardized rate of home care requests	O	
35. Percentage of old people benefiting from territorial care, with clinical and nursing assessment	C	
36. Average time (in days) between hospital or General Pratictioner (GP)’s reporting and first contact with the discharged patient	C	
37. Percentage of Saturday, Sunday, public holiday’s’ home visits	C	
38. Patients with a minimum of 8 accesses per month per 1000 resident rate	C	
39. Percentage of over 75 patients discharged that accessed the ED within 2 days	C	
40. Percentage of home assisted over 65 patients with Clinical Impairment Assessment (CIA) > 0.13 (clinical impairment assessment)	C	
41. Percentage of patients discharged and put in home-based care within 3 days	C	
42. Percentage of over 65 patients in home-based care and 2 hospitalization episodes	C	
43. Percentage of over 65 patients in home-based care and an ED access	C	
44. Rate of adult patients with home-based care and CIA on the total resident population	C	
45. Underage patients in home-based care with CIA/resident population rate	C	
46. Discharges with request of home-based care per 100,000 residents	H	
47. Rate of ED low urgency presentation, not generating hospitalization, standardized for sex and age per 1000 residents	O	
48. Residents with heart failure and at least 1 creatinine measurement	C	
49. Residents with heart failure and at least one sodium and potassium measurement	C	
50. Residents with heart failure and prescription of ACE inhibitors and sartans	C	
51. Residents with heart failure and prescription of beta blockers	C	
52. Residents with diabetes and at least one glycated hemoglobin measurement	C	
53. Residents with diabetes and at least 1 eye examination in the last two years	C	
54. Residents with stroke and a prescription for antithrombotic therapy	C	
55. Incidence of shoulder dystocia	H	
56. Incidence of postpartum hemorrhage	H	

^1^ Reference settings: O—organizational; H—hospital; C—community care; NH—nursing home.

**Figure A1:**
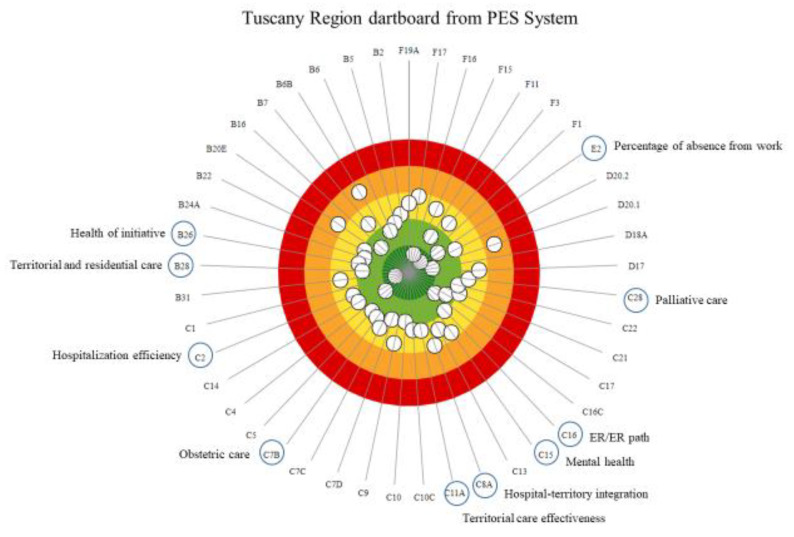
Tuscany Region dartboard.

**Figure A2:**
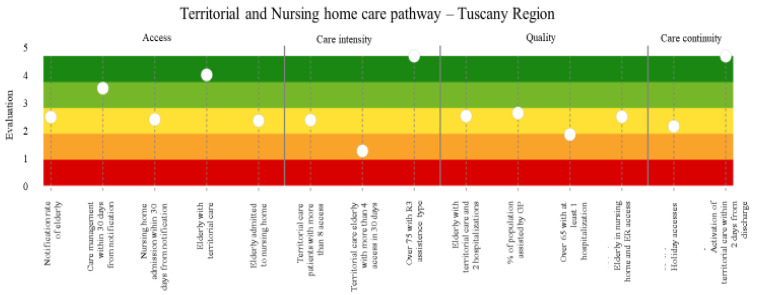
The pentagram of nursing home and home-delivered care pathway, Tuscany Region, 2018.

## Data Availability

Data concerning PES can be found at: https://performance.santannapisa.it/pes/start/start.php (accessed on 12 December 2021).

## Appendix A

**Table A1:** The COREQ Checklist, as reported in http://cdn.elsevier.com/promis_misc/ISSM_COREQ_Checklist.pdf (accessed on 12 December 2021) [48].

Section/Topic	Item No	Checklist Item	Reported on Page No
**Domain 1: Research Team and Reflexivity**			
Personal characteristics			
*Interviewer/facilitator*	1	Which author/s conducted the interview or focus group?Interviewer/facilitator	4
*Credentials*	2	What were the researcher’s credentials? E.g., Ph.D., MD	4
*Occupation*	3	What was their occupation at the time of the study?	1
*Gender*	4	Was the researcher male or female?	Not reported
*Experience and training*	5	What experience or training did the researcher have? Relationship with participants	4
Relationship with participants			
*Relationship established*	6	Was a relationship established prior to study commencement?	4
*Participant knowledge of the interviewer*	7	What did the participants know about the researcher? E.g., personal goals, reasons for doing the research	4
*Interviewer characteristics*	8	What characteristics were reported about the interviewer/facilitator? E.g., bias, assumptions, reasons, and interests in the research topic	4
**Domain 2: Study Design**			
Theoretical framework			
*Methodological orientation and theory*	9	What methodological orientation was stated to underpin the study? E.g., grounded theory, discourse analysis, ethnography, phenomenology, content analysis	3
Participant selection			
*Sampling*	10	How were participants selected? E.g., purposive, convenience, consecutive, snowball	4
*Method of approach*	11	How were participants approached? E.g., face-to-face, telephone, mail, email	4
*Sample size*	12	How many participants were in the study?	4
*Non-participation*	13	How many people refused to participate or dropped out? Reasons?	4
*Setting of data collection*	14	Where were the data collected? E.g., home, clinic, workplace	4
*Presence of non-participants*	15	Was anyone else present besides the participants and researchers?	N/A
*Description of sample*	16	What are the important characteristics of the sample? E.g., demographic data, date	4
Data collection			
*Interview guide*	17	Were questions, prompts, guides provided by the authors? Was it pilot tested?	N/A
*Repeat interviews*	18	Were repeat interviews carried out? If yes, how many?	N/A
*Audio/visual recording*	19	Did the research use audio or visual recording to collect the data?	4
*Field notes*	20	Were field notes made during and/or after the interview or focus group?	4
*Duration*	21	What was the duration of the interviews or focus group?	4
*Data saturation*	22	Was data saturation discussed?	N/A
*Transcripts returned*	23	Were transcripts returned to participants for comment and/or correction?	4
**Domain 3: Analysis and Findings**			
Data analysis			
*Number of data coders*	24	How many data coders coded the data?	4
*Description of the coding tree*	25	Did authors provide a description of the coding tree?	N/A
*Derivation of themes*	26	Were themes identified in advance or derived from the data?	4
*Software*	27	What software, if applicable, was used to manage the data?	N/A
*Participant checking*	28	Did participants provide feedback on the findings?	4
Reporting			
*Quotations presented*	29	Were participant quotations presented to illustrate the themes/findings? Was each quotation identified? E.g., participant number	N/A
*Data and findings consistent*	30	Was there consistency between the data presented and the findings?	4
*Clarity of major themes*	31	Were major themes clearly presented in the findings?	5/7
*Clarity of minor themes*	32	Is there a description of diverse cases or discussion of minor themes?	11/15

## Appendix B

### Appendix B.1. The National Database of Nursing Indicators (NDNQI^®^)

The National Database of Nursing Indicators (NDNQI^®^) is an American database that provides quarterly and annual reports on nursing indicators. NDNQI^®^ originated from the American Nursing Association (ANA) in 1998. Its main purpose is the systematic data collection of nursing quality indicators to measure nursing performance in a national context. This voluntary system compares more than 1100 USA health organizations until the unit level. It proposes traditional and operative measures (e.g., hours of nursing care per patient day, the prevalence of pressure ulcer, falls and falls with injury, nosocomial selective infection, and patient/family satisfaction with nursing care), includes staff mix measures (e.g., nurse-coworkers ratio), and it monitors policy and human resources management aspects (e.g., turnover, educational level, job satisfaction, work safety perception, nursing work index) regularly. NDNQI^®^’s novelty and foresight lie on dimensions as the decision-making process, nurse–physician relational aspects quality, personnel composition adequacy, work organization based on the presence of nursing models. As Lockhart [1] points out, the measures that this system provides are important because they allow hospitals not to be self-referential, that is to say that every organization can compare itself with other organizations at a national and regional level, and the measures are able to give information down to unit level.

### Appendix B.2. Collaborative Alliance for Nursing Outcomes (CaLNOC)

Collaborative Alliance for Nursing Outcomes (CaLNOC) started in 1996 in California as a no-profit initiative. CaLNOC provides performance measures on hospital quality trends allowing a start baseline for continuous benchmarking. As for the NDNQI^®^, adherence to CaLNOC is voluntary. The assessed indicators measure many dimensions as skill mix, nurse to patient ratio, voluntary turnover, nurse staff characteristics (e.g., educational level, work seniority), and the level of nurse process implementation (e.g., risk assessment, PICC use). The use of this measurement has been extended to other settings beyond acute care to include ambulatory care [2]. Benchmarking is the underlying factor that encourages healthcare systems to focus on the quality and efficiency of their outcomes. CalNOC is particularly valued for its capacity to support the responsiveness and strategic value of the data to members [3] since they have the possibility to use a customizable virtual dashboard that boosts its reporting capacity through making available selectable parameters of interest (ibidem).

### Appendix B.3. The Performance Evaluation System Experience (PES) and the Nursing Dimension

Figure A1 represents an example of the Tuscany Region dartboard, and the blue circles show some of the indicators considered in the proposal of nursing performance measurement integration inside the PES.

Indicators enriched with nursing dimension are circled in blue: percentage of absence from work, palliative care, ER, mental health, hospital-territory integration, territorial care effectiveness, obstetric care, hospitalization efficiency, territorial and residential care, and health of initiative (proactive health).

The dartboard is divided into five different bands, associated with different levels of performance: while the dark green centre indicated a high performance, the external red band indicates a very poor performance (dark green to red: excellent performance, good performance, average performance, poor performance, and very poor performance) according to the set standards [4]. The reference criteria for the positioning of performance are gathered from international standards—if existing, regional standards set out by the Regional Government or, if no measure is set, the “Regional mean” is used (standardized by all the factors that will allow a comparison hence a benchmarking among all the organizations) [5].

A graphic representation of a pentagram joins the dartboard, as shown in Figure A2.

Color bands are horizontal, framing the different stages of care pathways, and patients’ experience measures (PREMs and PROMs are continuously collected by the Observatory that is located inside the MeS Lab) are included and coexist with indicators gathered from administrative data, [6] and the notes represent performance indicators. While the dartboard refers to the provider of the service, the pentagram refers to the steps the patients take along the pathway, beyond organizational and physical boundaries crossing different care settings, and presents interactions with different subjects (different professionals) conjointly operating towards the same objective: the achieving of the best result possible for the patient [7,8]. To date, the clinical pathways represented through the pentagram are the oncological path, the mental health path, the maternal-infant path, the chronicity path, the emergency–urgency path, the home and residential care path for the elderly non-self-sufficient patients and the orthopaedic-traumatology path.
